# Traditional Chinese Acupressure Massage of the Quadriceps Femoris Can Relieve Flexion Pain after Undergoing Total Knee Arthroplasty

**DOI:** 10.1155/2022/1091174

**Published:** 2022-03-10

**Authors:** Zhiwei Fu, Changming Xu, You Wang, Xinhua Qu, Chunxi Yang

**Affiliations:** ^1^Department of Bone and Joint Surgery, Department of Orthopedics, Renji Hospital, School of Medicine, Shanghai Jiao Tong University, 145 Shandong Middle Road, Shanghai 200120, China; ^2^Department of Rehabilitation, Renji Hospital, School of Medicine, Shanghai Jiao Tong University, 160 Pujian Road, Shanghai 200001, China

## Abstract

**Objective:**

To reduce the pain of quadriceps during knee flexion after total knee arthroplasty and increase range motion of knee flexion.

**Design:**

Three-month prospective before/after quality improvement project. *Setting*. Department of Bone and Joint Surgery. *Participants*. A total of 80 patients who met the surgical indications were admitted to the outpatient department for surgery. They were randomly grouped by computer in advance, and the patients were divided into two groups according to the time of admission, each with 40 cases. *Intervention*. The intervention group performed routine rehabilitation exercises and received quadriceps acupoint massages for 20 minutes twice a day for two consecutive weeks. The control group performed routine rehabilitation exercises, such as gentle quadriceps massage for 20 minutes twice a day for two consecutive weeks. *Main Outcome Measures*. PPT (pressure pain threshold) of quadriceps femoris/VAS (visual analog scale) of knee flexion and motion of knee flexion.

**Results:**

The VAS score, range of motion, and tenderness threshold during flexion were significantly better in the intervention group than in the control group at 1, 2, and 4 weeks after surgery. But the VAS score, range of motion, and tenderness threshold did not significantly differ between groups at 12 weeks after surgery.

**Conclusion:**

Acupoint massage of the quadriceps femoris can relieve early flexion pain in patients after total knee arthroplasty. The trial was registered at clinical trials.gov.

## 1. Introduction

With the aging of the global population, an increasing number of patients have developed knee arthritis. Knee replacement surgery is considered to be the best treatment for end-stage knee arthritis. This surgery can effectively relieve pain and improve quality of life [1, 2]. Approximately 200,000 to 300,000 TKA are performed in China each year. Knee stiffness is one of the common complications after surgery, and its incidence is 5% to 7% [3–5].

Postoperative pain management plays an important role in the recovery of postoperative range of flexion motion, especially the recovery of joint range of motion within 2 to 3 weeks after TKA, which is of great significance for long-term postoperative rehabilitation [6–9]. There are still many patients with early postoperative flexion pain that causes joint stiffness. Kim et al. once used the French endermologie method to massage the quadriceps of patients after knee replacement and found that can relieve pain [10]. Self-massage by patients also can improve the range of flexion motion of the knee joint [11]. There are also some studies that used Thai massage or Swiss massage to treat patients with knee arthritis and showed that these types of massage are helpful for mobility and pain during knee flexion [12]. Traditional Chinese acupoint massage has been used for thousands of years in China. The meridians corresponding to the characteristics of the disease are located, and the appropriate acupoints on the meridians are manipulated. Among the acupoints, the Ashi points, Yanglingquan points, and Zusanli points are considered to be the key points for postoperative pain relief and muscle relaxation in patients who have undergone knee joint replacement [13–15].

This study mainly investigates whether the use of traditional Chinese acupoint massage can relieve pain around the quadriceps muscle and in the knee joint during flexion and extension exercises.

## 2. Materials and Methods

### 2.1. Subjects

Posters were used to recruit individuals planning to undergo knee arthroplasty, and those who were willing to participate were screened. The inclusion criteria were as follows: (1) preoperative knee arthritis severity score ≥ Kellgren–Lawrence III [16]; (2) a knee joint that is severely deformed, painful, and dysfunctional requiring primary unilateral TKA for end-stage OA; (3) an age between 50 and 80 years, without gender limitations; (4) active knee flexion range of motion before the operation of ≥90° with a flexion deformity or varus deformity magnitude of ≤25°; (5) normal liver and kidney function and electrocardiogram findings, without mental disorders or peripheral nerve diseases; and (6) the ability to think clearly, good comprehension, and having understood the purpose of the study.

The exclusion criteria were as follows: (1) according to the patient's intraoperative operation, the surgeon believes that routine rehabilitation should not be carried out after the operation. (2) If the patient is found to have osteoporosis during the operation, it is not suitable for early weight-bearing or fractures during the operation. (3) Patients with vision, hearing, and cognitive impairments. (4) Patients who cannot cooperate with follow-up on time due to their own reasons.

### 2.2. Procedure

Before the subjects were recruited, 1 to 80 numbers were randomly divided into the intervention group and the control group by the computer; the subjects were matched according to the sequence of 1–80 numbers according to the operation time, and the group where the numbers belonged was subject to the group the tester is in. All patients signed the written informed consent forms prior to enrollment. The bone and joint surgeries were started at our hospital. Prior to the study, this trial was approved by the medical ethics committee of Renji Hospital affiliated with Shanghai Jiaotong University School of Medicine (approval number: 2018-186). The trial was registered at clinical trials.gov (https://clinicaltrials.gov/ct2/show/ChiCTR1900022987).

The study was officially started in Renji hospital on March 20, 2019. The bone and joint surgeries were started at the hospital, and the subjects signed an informed consent form before participating in the experiment. A total of 80 patients undergoing unilateral total knee resurfacing from April 2019 to January 2020 were selected as the research subjects.

An orthopedic surgeon with 8 years of experience measured the quadriceps tenderness threshold, administered the VAS during flexion and WOMAC, and measured flexion range of motion for each included subject one week before surgery. On the first day after surgery, the same physical therapist with 3 years of work experience performed routine rehabilitation exercises for two weeks for both groups. After they were discharged, the participants performed home rehabilitation exercises and continued to communicate with the therapist every two days by phone or WeChat. The participants were followed up and provided rehabilitation guidance. Patients in the intervention group additionally received quadriceps acupoint massage for 20 min twice a day for 2 consecutive weeks.

**Figure 1:**
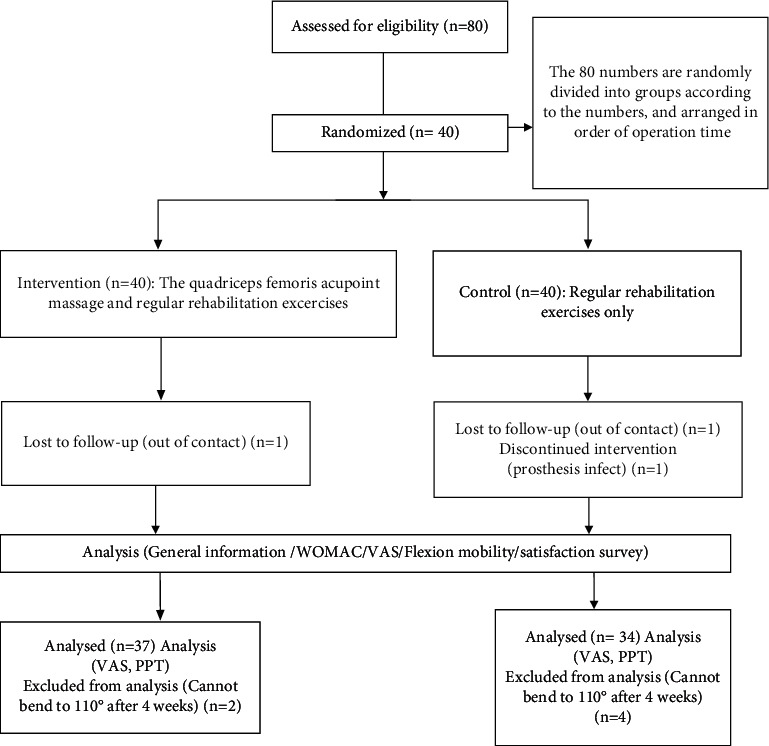
Patient selection process and study protocol.

During the whole process, one patient in the intervention group was out of follow-up, then the patient was eliminated (*n* = 39); in the control group, one patient was out of follow-up, one patient had a postoperative infection, and then the two were eliminated (*n* = 38). At 1, 2, 4, and 12 weeks after surgery, the patient's quadriceps tenderness threshold, VAS score during flexion, and ROM of flexion were measured. At 12 weeks after surgery, the subjects' WOMAC scores were measured. All assessments and measurements were performed by a physical therapist with 4 years of work experience. Because at four weeks postoperatively, 2 patients in the intervention group could not get the PPT value and VAS score because they could not flex to 110° and 3 patients in the control group could not flex to 110°. Therefore, in order to ensure the validity of the data results, they are removed when the PPT data and VAS score results are counted. The detailed process is shown in Figure 1.

### 2.3. Intervention

All 80 patients chose to undergo a median longitudinal incision of the knee joint and the medial parapatellar approach. Posteriorly stable knee joint prostheses were used in all patients. The patella was preserved during the operation, and a drainage tube was placed for 24 hours to heal the wound. The tourniquet was used to stop bleeding during the operation. The operation was performed by the same group of surgeons, and a standard analgesic method was used after the operation.

Both groups of patients were hospitalized for two weeks. The control group received ordinary quadriceps gentle massage and relaxation every day and twice a day, 30 minutes each for two weeks, respectively. The intervention group received quadriceps acupoint massage twice a day for two weeks, each for 30 minutes. Both sets of manual massages were performed by the same physical therapist. During hospitalization, the two groups of patients underwent routine rehabilitation exercises under the supervision of another physical therapist: quadriceps, gluteal muscle isometric contraction, and ankle pump exercises on the first day after surgery, passive and active knee flexion and extension training, quadriceps and gluteal muscle strength training, ground weight training, 20 minutes of cold compress around the knee joint after each training, and further knee joints on the second or third day after surgery, and mobility training, training of the muscles around the knee joint, weight-bearing training on the ground, and some targeted functional training according to the patient's condition after the third day after the operation. All patients were discharged from the hospital two weeks later, home rehabilitation videos were distributed, and the patients had weekly follow-up WeChat video chat with a physical therapist, until three months after surgery [17].

**Figure 2:**
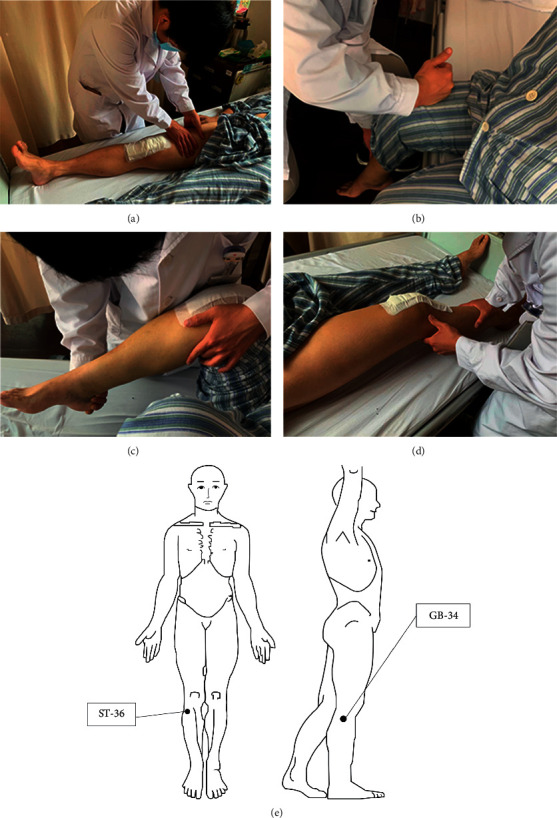
Traditional Chinese acupoint massage procedure.

The intervention group separately underwent quadriceps 20-minute acupoint massages [18–20] performed by the same physiotherapist twice a day as follows (Figure 2): first, at the massage and relaxation stage, a gentle massage was performed around the knee joint for 3–5 minutes. Second, at the acupoint pressing stage, identify the spasm and most painful point, Which called Ashi acupoint, and use thumb to press for 3 minutes. Fingers are used to identify the spasm point above his or her quadriceps femoris. Third, at the acupuncture stage to relax the tendons, the patient's Yanglingquan, Zusanli, and Ashi points were rolled in a relaxed sitting position, and each acupoint was addressed for one minute. Last, at the passive flexion stage, the therapist held the heel of the patient with one hand and the lower part of the popliteal fossa with the other hand, passively flexed the knee gently and slowly, gently pressed around the knee joint, and if there are points of spasm found in the process, gently pressed to relax.

## 3. Outcome Measures

### 3.1. Main Outcomes

According to previous clinical experience and literature [3, 4], the majority of patients after knee arthroplasty have a knee flexion range of about 60°–100° in the first week after surgery, a knee joint flexion range of about 80°–110° in two weeks after surgery, and the knee flexion range of motion around 90°–120° in the four weeks after surgery. Therefore, we started to measure the pain index (include PPT value and VAS) of knee flexion at 60° in the first week after surgery, then started to measure the pain index at 90° of knee flexion in the second week after surgery, and measured the knee at the fourth week after surgery. Measuring the pain index when the joint is flexed at 110°. If the subject cannot reach the measured angle within the corresponding time, in order to ensure the validity of the data, the pain index of the patient is directly eliminated.

#### 3.1.1. Pressure Pain Thresholds Test

**Figure 3:**
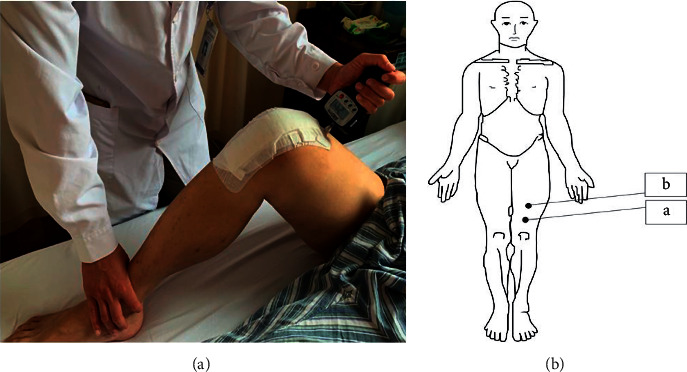
PPT was measured.

The FPX pain test algometer was used (Wagner, model: FPX50, specification: 50 1bf^*∗*^0.05 1bf, serial number: 18139, USA) [13, 21]. When the knee was flexed, a 1 cm^2^ rubber head was used to test the areas 5 cm and 10 cm from the upper pole of the patella. On the thigh, perpendicular to the skin, the handle was held firmly, and pressure was applied at a rate of 1 N/cm^2^/s until the subject felt pain for the first time and said “stop”; then, the value on the display was recorded. Three measurements were taken, and the average of the three measurements was used for analysis (Figure 3).

The PPT value was measured within 1 week before the operation and 1, 2, 4, and 12 weeks after the operation at 60°/90°/110° of knee flexion.

#### 3.1.2. VAS

The patients' VAS [22] pain scores were evaluated within 1 week before the operation and 1, 2, 4, and 12 weeks after the operation at 60°/90°/110° of knee flexion.

#### 3.1.3. Knee Joint Flexion Range of Motion Measurement

An orthopedic protractor was used to measure the range of flexion motion of the knee joint in the patients within 1 week before the operation and 1, 2, 4, and 12 weeks after the operation, and the maximum range of flexion motion without pain was measured.

### 3.2. Secondary Outcomes

#### 3.2.1. WOMAC Score

The WOMAC [23] was used. This scoring scale includes a total of 24 items related to 3 major aspects as follows: pain, stiffness, and joint function. The scale can objectively evaluate the patient's current functional state and physical ability. The WOMAC was administered within 1 week before the operation and 12 weeks after the operation.

## 4. Statistical Analysis

The SPSS 19.0 statistical software package was used for statistical analysis of the test results. The measurement data are expressed as the means ± standard deviation. To compare the data before and after the intervention, when the data were normally distributed, the paired sample *t-*test and repeated measures ANOVA (analysis of variance) were used; when the analysis of variance assumptions was not satisfied, the Friedman rank sum test was used. Comparisons between the two indicators were performed by the independent sample *t*-test. The chi-square test was used for enumeration data, and *P* < 0.05 was considered statistically significant.

## 5. Results

The first subject was recruited on May 14, 2019, the 80th subject was recruited on December 30, 2019, and the entire experiment ended on March 30, 2020. During the experiment, one subject from each group could not be contacted and was excluded from the experiment. One subject in the control group withdrew from the experiment because of a prosthesis infection. In the end, 39 people in the intervention group and 38 people in the control group completed the experiment. For the data analysis, the tenderness threshold and VAS score at 110° knee flexion could not be measured in 6 subjects because the knee joint could not be flexed to 110° at 4 weeks after surgery. To ensure the validity of the data, the PPT and VAS data of these 6 subjects (2 in the intervention group and 4 in the control group) were excluded.

### 5.1. Baseline Data

There were no significant differences in age, sex, height, weight, or body mass index (BMI) between the two groups of patients before surgery (*P* > 0.05) (Table 1).

### 5.2. Effects of Acupoint Massage

During the entire study period, all outcomes were measured by two physicians. These two physicians only participated in the measurement and recording of preoperative and postoperative outcomes with blinding to patient allocation.

#### 5.2.1. WOMAC Scores

The WOMAC score one week before the operation was 75.36 ± 17 in the intervention group and 74.87 ± 17.88 in the control group. There were no significant differences in the WOMAC score between the two groups (*P* > 0.05). The WOMAC score of the intervention group was 25.03 ± 11.16 at 3 months after surgery, and the WOMAC score of the control group was 29.84 ± 13.35 at 3 months after surgery. There were no significant differences in the WOMAC score between the two groups (*P* > 0.05) (Table 2).

#### 5.2.2. VAS Pain Score during Flexion

**Figure 4:**
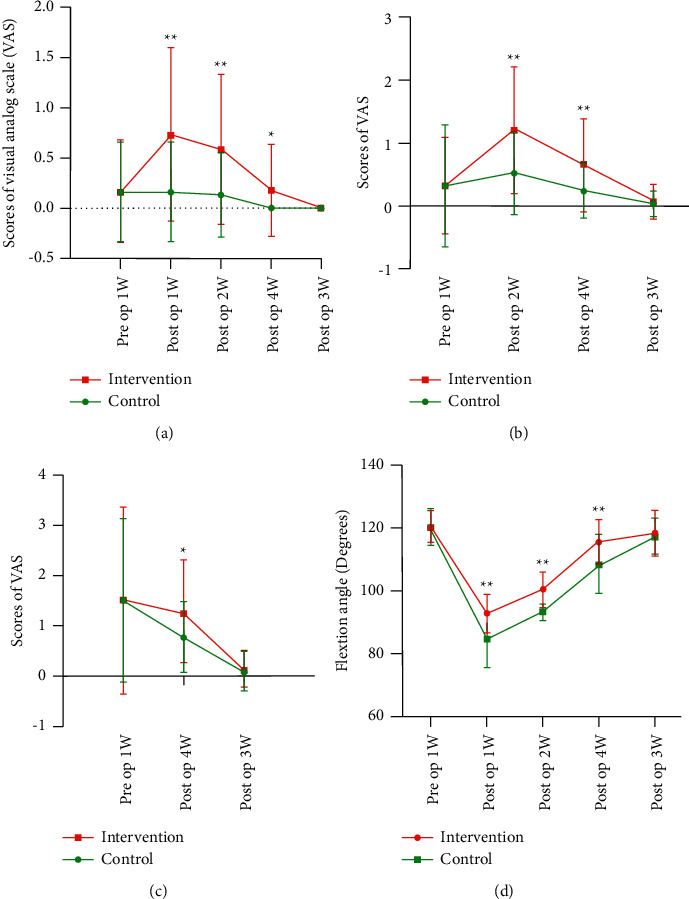
(a) Comparison of VAS scores between the two groups of patients with 60° flexion. (b) Comparison of VAS scores between the two groups of patients with 90° flexion. (c) Comparison of VAS scores between the two groups of patients with 110° flexion. (d) Comparison of flexion mobility between the two groups of patients.

Regarding the postoperative VAS score during knee flexion, the intervention group was statistically significantly lower than that of the control group at 1 week, 2 weeks, and 4 weeks after surgery (*P* < 0.05), but there were no significant differences between the two groups at 12 weeks after surgery (*P* > 0.05) (Figures 4(a)–4(c)).

#### 5.2.3. Flexion Mobility

Regarding postoperative knee flexion range of motion, there were no significant differences between the two groups of patients one week before surgery (*P* > 0.05); the intervention group was had a significantly larger range of motion than did the control group at 1, 2, and 4 weeks after surgery (*P* < 0.05), but there were no significant differences between the two groups at 12 weeks after surgery (*P* > 0.05) (Figure 4(d)).

#### 5.2.4. Pressure Pain Threshold

**Figure 5:**
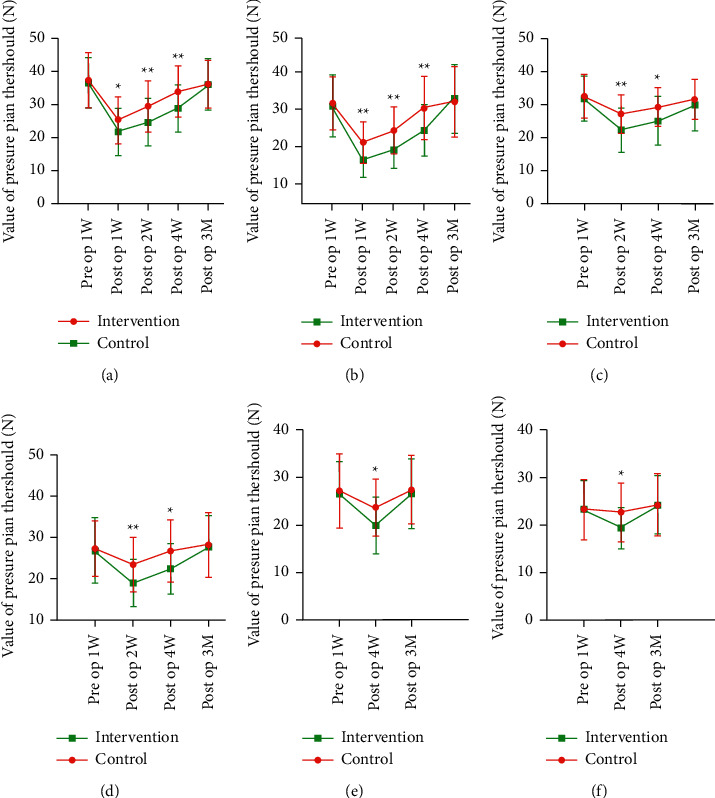
Comparison of the PPT values between the two groups of patients at different degrees of flexion. (a) Comparison of PPT values of A point between two groups of patients with the knee at a flexion of 60°. (b) Comparison of PPT values of B point between two groups of patients with the knee at a flexion of 60°. (c) Comparison of PPT values of A point between two groups of patients with the knee at a flexion of 90°. (d) Comparison of PPT values of B point between two groups of patients with the knee at a flexion of 90°.

The PPT values measured postoperatively at the front and middle of quadriceps femoris at knee flexion had statistically significantly improved in the intervention group at 1 week, 2 weeks, and 4 weeks after the operation (*P* < 0.05). However, there were no significant differences between the two groups at 12 weeks after the operation (*P* > 0.05), as shown in Figure 5.

## 6. Discussion

Flexion pain after knee replacement may occur for the following reasons. First, due to postoperative traumatic inflammatory reactions and joint edema, the skin tension increases, nerves innervating the skin and muscles around joints become sensitized, and flexion of the knee joint causes the sutured wound to stretch, inducing pain and preventing the patient from being able to flex the joint. Second, the phenomenon of reflex muscle guarding may occur. The quadriceps cramps response is caused by pain factor stimulation or early postoperative local tissue injury, especially when the knee joint is flexed, and the quadriceps spasm causes resistance [24, 25]. Third, endogenous quadriceps spasm is a reflex muscle contraction triggered by pain caused by direct or indirect trauma or inflammation. Activation of the sympathetic nervous system, emotional stress, cold stimulation, and braking of the tissues may all cause muscle reflex contractions. These contractions restrict joint movement, leading to tissue ischemia, circulatory arrest, and metabolite retention, thereby stimulating nerve endings, inducing or aggravating pain, and causing spastic self-circulation [24, 26].

In previous studies performed by other scholars, traditional Chinese acupoint massage with Chinese massage techniques has been shown to release biologically active chemical substances from the nervous system, tissues, and organs, which can improve blood circulation and accelerate the elimination of inflammatory and pain-causing substances and acidic metabolites, thereby producing therapeutic and analgesic effects [27]. The techniques act at different levels of the nervous system. At the peripheral level, TCM massage techniques can increase the amount of hypothalamic endorphins, reduce the amount of inflammatory mediators such as bradykinin and serotonin, and promote the absorption of edema inside and outside the nerve roots to reduce inflammation, yielding an analgesic effect. At the spinal cord level, the stimulation produced by massage techniques can weaken T-cell activity and close the gate of spinal pain impulse transmission. At the spinal cord's upper central level, pain signals may interact with signals generated by massage, suppressing the pain impulse and yielding an analgesic effect. Regarding the psychological mechanism of pain, manual stimulation can adjust the patient's mental state, reduce the amount of pain-causing substances in the brain, thereby reducing the severity of pain [28]. In addition, regarding the massage of acupoints in traditional Chinese medicine, one of the acupoints is called Ashi, and it is the most painful point around the knee joint that we often call the trigger point, a type of focal intramuscular tenderness and muscle induration. Band tightness may be caused by muscle fibers that are damaged by trauma and cause contractures to form trigger points. Due to the generation of trigger points, muscle cramps and pain develop, which affect the flexion range of motion of the knee joint. We use the fingers in acupoint massage to loosen this induration [29–31].

Some scholars have performed dry needle techniques in patients undergoing knee replacement surgery by physically stimulating the pain points around the quadriceps muscle of the patient, and the results show that this method can increase the mobility of the patient and reduce the severity of pain. The authors of another study have suggested that “the dry needle technique may induce the liberation of endogenous opioid peptides, favoring tissue regeneration and reducing the concentration of nociceptive and sensitive chemical substances in the immediate environment around the trigger point” [32]. Acupoint massage with Chinese medicine uses the same method of physical stimulation for treatment [33, 34].

This study is about the treatment of the quadriceps femoris after knee arthroplasty. After two consecutive weeks of hand massage during hospitalization, the VAS score of the intervention group during knee flexion was significantly lower than that of the control group at 1, 2, and 4 weeks after the operation. In the upper quadriceps femoris, the tenderness threshold also increased to a certain extent, which reduced the pain sensitization of the skin and muscles around the knee joint. Due to the massage, it is obvious that the patient's quadriceps muscles gradually become softer. During knee joint flexion, the quadriceps muscles had significantly less tension, and the spasms were relieved. Although there were no significant differences in the indicators between the two groups of patients at three months after the operation, the pain severity had significantly decreased.

## 7. Limitations

Due to insufficient sample size or too short intervention time, there was no significant statistical difference in the indicators of the two groups of subjects after three months. For patients with poor postoperative functional recovery, other treatments may be given two weeks after discharge to promote the range of flexion.

## 8. Conclusion

Traditional Chinese acupoint massage of the quadriceps can reduce the severity of pain around the knee joint to a certain extent in the early stage, relieve spasms of the quadriceps muscle during knee flexion, reduce the severity of pain during flexion, and improve the range of motion of the knee joint. The treatment can also improve patient satisfaction during postoperative rehabilitation.

## Figures and Tables

**Table 1:** Comparison of the basic information of the two groups of patients.

Group	Num	Age	Man	Female	Height (cm)	Weight (kg)	BMI
Intervention	39	67.05 ± 7.27	6 (15.4%)	33 (84.6%)	158.77 ± 5.54	66.13 ± 9.58	26.22 ± 3.57
Control	38	66.53 ± 8.09	9 (23.7%)	27 (76.3%)	160.32 ± 6.82	65.82 ± 9.03	25.60 ± 3.31
*P*-value			0.401		0.278	0.883	0.436

**Table 2:** Comparison of the WOMAC scores in the prospective patients.

Group	*N*	One week before the operation	Three months after the operation
Intervention	39	75.36 ± 17.24	25.03 ± 11.16
Control	38	74.87 ± 17.88	29.84 ± 13.35
*P*-value		0.903	0.090

## Data Availability

The data are available upon request to the corresponding author.

## Abbreviations

PPT: Pressure pain threshold
VAS: Visual analog scale
TKA: Total knee arthroplasty
WOMAC: Western Ontario and Mcmaster Universities Arthritis Index.
